# Azido­{1-[2-(dimethyl­amino)­ethyl­iminometh­yl]naphthalenolato}copper(II). Erratum

**DOI:** 10.1107/S2056989016002139

**Published:** 2016-08-31

**Authors:** Qi-Yong Zhu, Yi-Jun Wei, Feng-Wu Wang

**Affiliations:** a Department of Chemistry, Huainan Normal College, Huainan 232001, People’s Republic of China

## Abstract

In the paper by Zhu *et al.* [*Acta Cryst.* (2006), E**62**, m983–m985], the metal atom was reported incorrectly.

## Abstract 2

Erratum to *Acta Cryst.* (2006), E**62**, m983–m985.

The authors of Zhu *et al.* (2006) ▸ have indicated that the metal atom in the reported complex was assigned incorrectly. The paper is therefore withdrawn from the published literature and associated databases.

## References

[bb1] Zhu, Q.-Y., Wei, Y.-J. & Wang, F.-W. (2006). *Acta Cryst.* E**62**, m983–m985.

